# Hepatoprotective Effect of Corm of *Ensete ventricosum* (Welw.) Cheesman Extract against Isoniazid and Rifampicin Induced Hepatotoxicity in Swiss Albino Mice

**DOI:** 10.1155/2021/4760455

**Published:** 2021-08-13

**Authors:** Abebe Dukessa Dubiwak, Tesaka Wondimnew Damtew, Mengistu Welde Senbetu, Delenasaw Yewhalaw, Tsegaye Girma Asere, Gebi Nemo, Minale Fekadie Baye

**Affiliations:** ^1^Division of Medical Biochemistry, Department of Biomedical Sciences, Institute of Health Sciences, Jimma University, Jimma, Ethiopia; ^2^Department of Medical Laboratory Sciences and Pathology, College of Health Sciences, Institute of Health Sciences, Jimma University, Jimma, Ethiopia; ^3^Department of Chemistry, College of Natural Sciences, Jimma University, Jimma, Ethiopia; ^4^Department of Pathology, Institute of Health Sciences, Jimma University, Jimma, Ethiopia

## Abstract

Drug-induced liver injury (DILI) is one of the cumbersome health-related problems which render approximately 50% of liver failure and patients to receiving liver transplantation every year. Antituberculosis drugs such as isoniazid and rifampicin are potentially rendering hepatotoxicity. *Ensete ventricosum* (Welw.) Cheesman is an herbaceous perennial plant that contributes to the indigenous ethnomedicinal values for the society. This study aimed to investigate the hepatoprotective effect of corm of *Ensete ventricosum* (Welw.) Cheesman extracts against isoniazid and rifampicin induced hepatotoxicity in Swiss albino mice. The study was conducted on 30 Swiss albino mice randomly allocated into five groups. Group I, group II, group III, group IV, and group V were the groups in which mice were given distilled water, only isoniazid and rifampicin, isoniazid and rifampicin along with 200 mg/kg corm of *Ensete ventricosum* (Welw.) Cheesman extract, isoniazid and rifampicin along with 400 mg/kg corm of *Ensete ventricosum* (Welw.) Cheesman extract, and isoniazid and rifampicin along with silymarin per oral per day, respectively. On the 30th day of the experiment, mice were sacrificed after anesthetized, and blood was drawn for the liver function test, and the liver was also taken from each experimental mouse for histopathological evaluation. Data were entered into EpiData version 3.1 subsequently exported to SPSS version 25 for analysis by using one-way ANOVA. Plasma alanine aminotransferase (ALT) levels, aspartate aminotransferase (AST), alkaline phosphatase (ALP), and total bilirubin (TBIL) of group II mice were significantly (*p* < 0.05) elevated as compared to group I. The group of mice treated with a corm of *Ensete ventricosum* (Welw.) Cheesman at a dose of 400 mg/kg (group IV) and silymarin100 mg/kg (group V) showed a significant (*p* < 0.05) decrease in ALT, AST, ALP, and TBIL as compared to the group II. The liver section of group II showed a change in liver architecture; however, these deformities were not noticed in group IV mice. The result showed corm of *Ensete ventricosum* (Welw.) Cheesman extract has a very promising hepatoprotective potential against isoniazid and rifampicin induced liver injury.

## 1. Introduction

Hepatotoxicity is one of the cumbersome complications that can be mainly caused by overdoses of certain medicinal drugs, industrial chemicals, even dietary supplements, and excess consumption of alcohol [1, 2].

Being the liver is the principal organ in the drug metabolism, exposes the organ to toxic injury and makes it the most frequently targeted organ in drug toxicity [3, 4]. The most frequent hepatotoxic drug reactions evoke moderate-to-severe injury to hepatocytes [5, 6]. Biochemical indicators of hepatocellular injury are a rise of liver biomarkers such as aspartate transaminase (AST), alanine transaminase (ALT), alkaline phosphatase (ALP), and total bilirubin (TBIL) [7, 8].

The usual drugs associated with hepatotoxicity are antituberculosis (anti-TB) drugs, which are utilized to treat tuberculosis disease [9]. Isoniazid (INH) and rifampicin (RIF) are anti-TB drugs that have been treating TB-infected individuals. They are metabolized in the liver and the principal agents responsible for anti-TB drug-induced hepatotoxicity [9, 10].

INH and its metabolites (such as hydrazine) are associated with serious hepatotoxicity and potentially fatal liver injury [11, 12]. INH also induced hepatotoxicity via generating ROS that leads to oxidative stress. Hydrazine can generate oxygen radicals or superoxide and the isoniazid treatment increment of superoxide [11].

Patients on isoniazid coadministration with rifampicin therapy have increased hepatotoxicity [13]. In a meta-analysis, it was revealed that rifampicin increased the prevalence of drug-induced liver injury in a multidrug regimen from 1.6% to 2.55% in adults [14]. Hepatotoxicity attributed to anti-TB drugs has been reported 5–34.9% in people treated with anti-TB drugs [15, 16].

Antituberculosis drugs induced hepatotoxicity (anti-TB DIH) is a major problem in HIV coinfection TB immunocompromised in Ethiopia [17]. A study performed in Ayder Referral Hospital of Tigray regional state showed that TB/HIV coinfected patients treated with antituberculosis developed grade one hepatotoxicity and severe hepatotoxicity (≥grade 2) 45% and 15%, respectively [18]. In Jimma Medical Center in 2013, the incidence of anti-TB drug-induced hepatotoxicity was 11.5% [19].

*Ensete ventricosum* (Welw.) Cheesman belongs to the monocotyledonous and monocarpic family *Musaceae* and is an herbaceous perennial plant [20]. It is the source of more than 10 minerals which include calcium (Ca), sodium (Na), potassium (K), magnesium (Mg), iron (Fe), manganese (Mn), copper (Cu), and zinc (Zn) [20, 21].

Besides, to use it as a staple and costaple food for millions of Ethiopians contributes to indigenous ethnomedicinal values of the society. *Ensete ventricosum* (Welw.) Cheesman is used in traditional for treating liver diseases, expel of the placenta, treatment of cough, dysentery, healing of the bone fracture, diabetes, kidney stone, and dysuria, and antimicrobial activity against viral, bacterial, fungal, and nematode diseases of humans [22–24]. Several minerals and phytochemicals in the extract corm of the *Ensete* genus have the capability of the cell-protective effect via scavenging free radicals and boosting the antioxidant defense system (AODS) [21, 25–28], but no study is performed on its hepatoprotective effect yet. Therefore, this study had revealed a hepatoprotective effect of the corm of *Ensete ventricosum* (Welw.) Cheesman against hepatotoxicity induced by anti-TB drugs.

## 2. Materials and Methods

### 2.1. Plant Collection and Authentication

*Ensete ventricosum* (Welw.) Cheesman was obtained from Oromia region, Southwest Shoa, Wanci woreda, and then subsequently authenticated by plant taxonomist at Addis Ababa University National Herbarium, and AD-001/2020 voucher number was given, and the plant leaf was deposited at the herbarium for further reference.

### 2.2. Plant Extraction

The corm of *Ensete ventricosum* (Welw.) Cheesman is the underground part of *Ensete ventricosum* (Welw.) Cheesman. It was dug out, and the corm was uprooted from the soil, the root was removed from it, and chopped into small pieces. It was air-dried under a shaded area at room temperature. The gross weight of the chopped corm of *Ensete* was 400 g which was then ground into coarse powder and macerated by using 80% methanol.

The extract was filtered, and then, methanol solution was evaporated by a rotary evaporator to have a solid consistency and dried by a freeze dryer (lyophilizer). Finally, residue extract was packed in air-tight glass bottles with proper labels and kept in a refrigerator at 4°C until used for the experiment.

### 2.3. Preliminary Phytochemical Screening of the Extract

Testing for alkaloids, terpenoid, flavonoid, phenol, steroid, quinone, saponin, tannin, and glycoside of hydromethanolic extract of corm of *Ensete ventricosum* (Welw.) Cheesman was performed by following procedures obtained from previous studies [29].

### 2.4. Experimental Animals

A total of 30 either sexes, 8–10 weeks old, and weighing 30–41 g Swiss albino mice were obtained from Jimma University Tropical and Infectious Disease Research Center (JUTIDRC), Sokoru, Jimma, Ethiopia. The experimental animals were allowed to acclimatized laboratory conditions at Jimma University College of Agriculture and Veterinary Medicine (JUCAVM), postgraduate Veterinary Medicine Laboratory, for two weeks before the experiment was commenced. Standard food pellets (*ad libitum*) and tap water were supplied for animals at all times. The animals were maintained on a 12 h light/dark cycle in an ambient temperature (20–25°C) and humidity environment. At the end of two weeks, experimental animals were randomly grouped into 5 groups each group consisting of 6 mice.

### 2.5. Drug Dose for Inducing Hepatotoxicity

The INH and RIF doses for inducing liver damage were 75 mg/kg and 150 mg/kg administered per oral (PO) by gavage, respectively [30].

### 2.6. Grouping of Experimental Animals and Treatment Protocol

The doses of INH and RIF to induce hepatotoxicity were used from previous studies [30], and the initial dose of the corm of *Ensete ventricosum* (Welw.) Cheesman extract was 10% of 2000 mg/kg because an extract of *Ensete* had no toxicity effect up to 2000 mg/kg [28]; then, the second dose was two folds of the initial dose which was 20% of 2000 mg/kg (400 mg/kg) according to OECD guidelines.

1. Group I (normal control group): representing mice received 1 ml/kg of distilled water PO daily for thirty days
2. Group II (negative control): representing mice received INH 75 mg/kg plus RIF 150 mg/kg PO daily for thirty days
3. Group III (experimental): representing mice received corm of *Ensete ventricosum* (Welw.) Cheesman extract 200 mg/kg and INH 75 mg/kg plus RIF 150 mg/kg PO daily for thirty days
4. Group IV (experimental): representing mice received corm of *Ensete ventricosum* (Welw.) Cheesman extract 400 mg/kg and INH 75 mg/kg plus RIF 150 mg/kg PO daily for thirty days
5. Group V (silymarin control): representing mice received silymarin 100 mg/kg and INH 75 mg/kg plus RIF 150 mg/kg PO daily for thirty days [31]

### 2.7. Bodyweight and Liver Index (Liver/Bodyweight %)

The bodyweight of the mice was measured weekly to see bodyweight change in all groups of experimental animals. The liver index was calculated as the liver weight of each mouse divided by their respective bodyweight multiplied by 100 (g/g).

### 2.8. Blood Collection and Serum Preparation from Mice

On the 30^th^ day, the mice were fasted overnight and anesthetized by 100 mg/kg ketamine/12.5 mg/kg xylazine injection. The mice were euthanized by cervical dislocation after cardiac puncture for blood collection. About 2–2.5 ml of blood collected from each mouse was placed in a serum separating test tube and left for 30 minutes at room temperature to clot. The serum was separated using a micropipette (1000 *µ*l) after centrifugation with the speed of 3000 rpm at room temperature for 10 minutes, and isolated serum was stored at −20°C until serum was analyzed for the liver biomarker.

### 2.9. Biochemicals Analysis

From serum, the liver damage biomarkers are the ALT, AST, ALP, and TBIL analyzer according to the standard principles and procedures of the kit manufacturer manual.

### 2.10. Histopathological Study

The liver tissue was taken from each mouse carefully after the mice were sacrificed via dissection from the neck to the pubis and opened the peritoneum by using a sterile surgical blade. The liver tissue was preserved by 10% of buffered neutral formalin in saline. After the tissues were processed and embedded in paraffin wax, 5 mm thick sections were taken and stained with hematoxylin and eosin for histopathological examination.

### 2.11. Statistical Tests

The protective effect of the intervention among experimental groups of animals was done by using SPSS statistical software package version 25. To evaluate mean differences of various parameters between the groups use one-way ANOVA, followed by post hoc. Tukey and *p* value less than 0.05 were considered statistically significant. The microscopic evaluation was a qualitative analysis carried out by a senior pathologist through preparing microscopic slides for each group and presented in the form of photomicrography.

### 2.12. Ethical Approval

This study was performed after the ethical clearance letter obtained from the Research and Ethical Review Committee of the Institute of Health, Jimma University, with a protocol number of Ref.No. IHRPGD/714/2020. The uses of animals and all activities in this experimental study were carried out according to the regulation of animal care and use of JUCAVM.

## 3. Results

### 3.1. Result of the Phytochemical Screening Test

The result of phytochemical screening of the hydromethanolic extract of the corm of *Ensete ventricosum* (Welw.) Cheesman showed the presence of bioactive constituents such as alkaloid, flavonoid, steroid, quinone, saponin tannin, and glycosides (Table 1).

### 3.2. Bodyweight of the Mice

**Figure 1:**
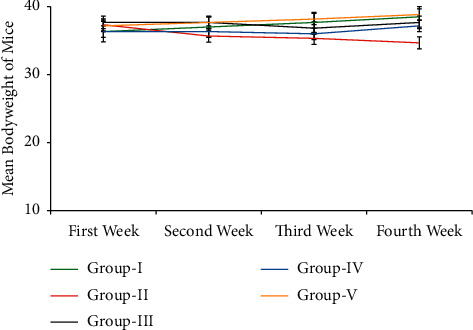
The mean bodyweight of the Swiss albino mice from the first week to the fourth week (the results are expressed as mean ± SE).

The initial mean weight of the mice was 37.00 ± 2.60 g (30 to 41 g). Between all groups, the mean bodyweight of the mice at 1^st^, 2^nd^, 3^rd^ and 4^th^ week were insignificant differences (*p* > 0.05), despite group II mean bodyweight were numerical decrement from the first week to the fourth week (Figure 1) when compared with the mean bodyweight of group I.

### 3.3. Effect of Corm of *Ensete ventricosum* (Welw.) Cheesman Extract on Liver Enzyme Parameters and Serum Total Bilirubin in Isoniazid and Rifampicin Challenged Mice

The effect of corm of *Ensete ventricosum* (Welw.) Cheesman extract on liver enzyme parameters and serum total bilirubin (TBIL) in isoniazid and rifampicin challenged mice is given in Table 2.

### 3.4. Effect of Corm of *Ensete ventricosum* (Welw.) Cheesman Extract on Liver Index (Liver/Bodyweight %)

The liver index in different experimental groups of mice is given in Table 3.

### 3.5. Liver Histopathological Findings

**Figure 2:**
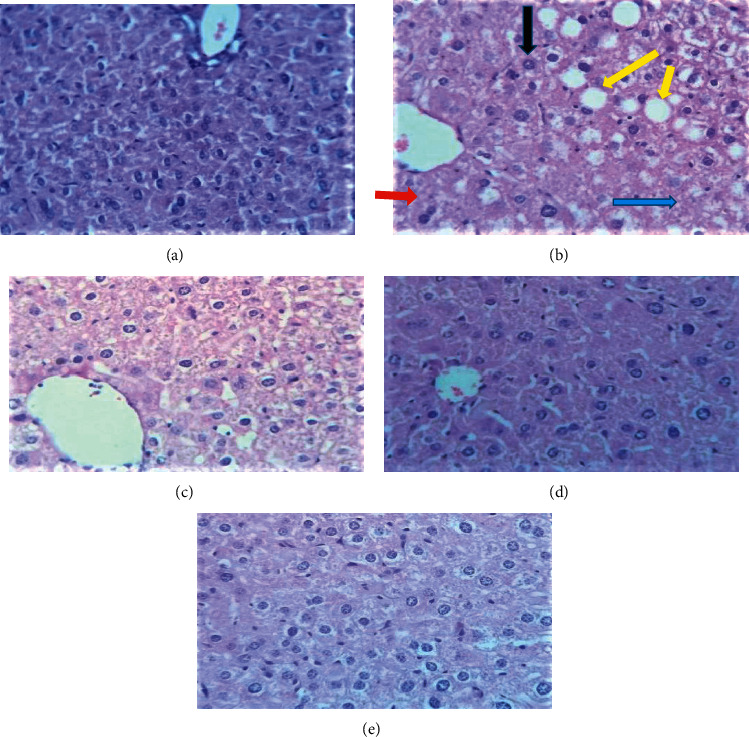
Photomicrograph of the liver of experimental animals (stained with hematoxylin and eosin). Group I (a); group II (b); group III (c), group IV (d), and group V (e). Red arrow, hypertrophy; black arrow, vacuolar change; yellow arrow, fatty change, and blue arrow inflammatory infiltration.

Microscopic examination of the liver sections of mice showed a visible difference in the liver architecture between the controls and the treatment groups. As we can observe from the microscopic slide, in the normal control group of mice, the liver section showed normal hepatic parenchyma composed of the plate of hepatocytes, central vein, and portal tracts with portal veins, hepatic vessels, and bile ducts (Figure 2(a)). In contrast, the liver section slide of the isoniazid and rifampicin only treated group showed hepatic parenchymal composed of hepatocyte with hypertrophy, mild-moderate vacuolar degeneration, mild necrosis, fatty change, and mild mixed inflammation cells infiltration throughout parenchymal including the portal area (Figure 2(b)). However, the group of mice treated orally with the corm of *Ensete ventricosum* (Welw.) Cheesman extract significantly regenerated the liver architecture in isoniazid and rifampicin treated mice, and a dose-dependent difference in regenerative capacity was observed between group III (200 mg/kg) and group IV (400 mg/kg). The silymarin treatment also significantly regenerated the liver architecture in isoniazid and rifampicin treated mice (silymarin control) compared to only the isoniazid and rifampicin treated group, eventhough there is minor infiltration.

## 4. Discussion and Conclusion

A serious adverse drug reaction of the antituberculosis treatment is hepatotoxicity [9]. In the present study, after daily administration of the INH 75 mg/kg and RIF 150 mg/kg) for 30 days, the hepatotoxicity was confirmed by significant elevation of the serum level of liver enzymes such as ALT (*p* < 0.01), ALP (*p* < 0.01), and AST (*p* < 0.01) levels in the mice orally administered of INH and RIF (group II) as compared to the mice orally administered with distilled water (Table 2). Meanwhile, an obvious death of hepatocytes, accompanied by mild necrosis, inflammatory infiltration, mild-moderate vacuolar change with vacuolar degeneration, and fatty degeneration throughout all hepatic parenchymal was also observed in the liver section of mice treated with only INH and RIF (Figure 2(b)). This finding is in harmony with the previous studies done by Shabbir et al. [16], Chen et al. [30], and Siddique et al. [32].

The plausible explanation of the INH and RIF that causes hepatocellular damage and attribute the elevation of the liver enzymes level in the serum is during reactive metabolites of those drugs covalently bound macromolecules of the cell (hepatocyte), resulting in depriving cellular integrity and liver enzymes released from the cell to the blood [33, 34]. Additionally, these anti-TB drugs could cause cellular damage through the induction of oxidative stress as a consequence of the depletion of glutathione (GSH)/glutathione peroxidase (GPx), superoxide dismutase (SOD), and catalase (CAT) levels within hepatocytes via activation of CYP2E1 [33–35].

Treatment with hydromethanolic extract of corm of *Ensete ventricosum* (Welw.) Cheesman restored the serum level of liver enzymes near to normal in the INH and RIF challenged mice. The corm of *Ensete ventricosum* (Welw.) Cheesman extract treatment at a dose of 200 mg/kg (group III) showed a significant reduction (*p* < 0.05) of ALT, AST, and ALP levels in the INH and RIF treated mice as compared to only the INH and RIF treated group (group II). Moreover, treatment at a higher dose (400 mg/kg) lowered the level of liver enzymes near to normal (*p* < 0.01) in the INH and RIF treated mice as compared to the INH and RIF only treated group. Treatment with silymarin (group V) also showed a significant reduction (*p* < 0.01) in the level of liver enzymes in INH and RIF treated mice compared to the INH and RIF only treated group (Table 2).

Other studies were performed on the hepatoprotective effect of the *Musaceae* family, like fruit pulp extract of *Musa paradisiaca* and stems of *Musa sapientum* Linn. against carbon tetrachloride-induced in rats similar to our finding [36, 37]. This may be due to synergistic activities of phytochemicals in the hydromethanolic corm of *Ensete ventricosum* (Welw.) Cheesman extract which have the capability to decreasing CYP2E1 enzymatic activity of alkaloid [38], antioxidative, and free radical scavenging properties of tannin, important in protecting cellular oxidative damage including lipid peroxidation [39, 40], antioxidant action by enzyme zinc-dependent superoxide dismutase [21, 32], hepatoprotective activity via modulation of its antioxidant of saponin [41, 42], and flavonoid [43].

INH and RIF only treated group of mice (group II) had statistically significant (*p* < 0.01) increment of total bilirubin as compared to a normal control group of mice (group I) (Table 2). This finding was in line with the study performed by Naji et al. [34] and Chandra and Shanmugapandivan [35]. This might be due to the combination of INH and RIF resulted in a higher rate of inhibition hepatic clearance of bile, an increase in lipid peroxidation in the hepatocyte, and cytochrome P450 involved in the synergistic effect of RIF and INH [34, 35, 44].

Treatment with the corm of *Ensete ventricosum* (Welw.) Cheesman extract lowered serum total bilirubin in INH and RIF treated mice. The mice treated with INH and RIF plus corm of *Ensete ventricosum* (Welw.) Cheesman extract at a dose of 400 mg/kg significantly reduced (*p* < 0.05) as compared to INH and RIF only treated mice (Table 2). This result has consistency with studies performed on the hepatoprotective effect against INH and RIF induced hepatotoxicity of *Cassia fistula* leaves by Ilyas et al. [45] and *Eclipta alba* by Chandra and Shanmugapandivan [35]. This might be due to the presence of secondary metabolites that render to restore injured hepatocytes such as flavonoids, steroids, tannins, saponins, and alkaloids. The corm of the *Ensete ventricosum* (Welw.) Cheesman also consists of those secondary metabolites. Moreover, a significant decrease (*p* < 0.01) total bilirubin level was observed in the silymarin (100 mg/kg) treated mice as compared to only the INH and RIF treated mice.

Furthermore, the present study pointed out a notable (*p* < 0.01) increased in the liver index in coadministration of INH with RF mice (group I) (Table 3). This finding is consistent with earlier study done by Tilaye et al. [46]. Also, this result revealed that corm of *Ensete ventricosum* (Welw.) Cheesman extract at 400 mg/kg (group IV) causes a significant (*p* < 0.01) decrement of the liver index in INH and RIF treated groups as compared to only INH and RIF treated group (Table 3). Silymarin treated group also has a comparable result. This is probably due to the healing of the liver to perform all its physiological functions including burning the fat it had accumulated inside its cells.

Microscopic examination of the liver sections of mice showed a visible difference in the liver architecture between the controls and the treatment groups (Figures 2(a)–2(e)). The liver section slide of the INH and RIF only treated group showed hepatic parenchymal composed of hepatocyte with hypertrophy of hepatocytes in the central area of the lobule, moderate vacuolar change, and vacuolar degeneration around the portal area, with mild inflammatory cell infiltration throughout parenchymal includes portal area and fatty change (Figure 2(b)).

The present finding supported by the previous studies Liu et al. [8] reported on bile, lipid, and purine metabolism involved in the hepatotoxicity of first-line antituberculosis drugs, Huang et al. [47] reported rifampicin induced hepatic lipid accumulation, and Dong et al. Wali et al. [48], Evan et al. [33], and Bais and Saiju [49] reported isoniazid plus rifampicin induced liver injury in mice. It is reasonable to assume that rifampicin-activated PXR is involved in the upregulation of genes for fatty acid synthesis. In addition to hepatic de novo lipogenesis, the increased uptake of free fatty acids from circulation to the liver plays an important role in the development of hepatic lipid accumulation [47].

However, the group of mice treated orally with the corm of *Ensete ventricosum* (Welw.) Cheesman extracts significantly regenerated the liver architecture in INH and RIF treated mice, and a dose-dependent difference in regenerative capacity was observed between group III and group IV (Figures 2(c) and 2(d)). This perhaps is due to the antihyperlipidemic effect of glycosides, antioxidant effect of saponins, and anti-inflammatory effect of flavonoids [50] in the corm of *Ensete ventricosum* (Welw.) Cheesman.

The silymarin treatment also significantly regenerated the liver architecture in INH and RIF treated mice (silymarin control) (Figure 2(e)) compared to only the INH and RIF treated group (Figure 2(b)), eventhough there is minor infiltration. Maybe due to it inhibits several isoforms of CYT P450 enzymes, potentiates the antioxidant capacity of the liver, acts as a scavenger of oxygen free radicals, inhibits the synthesis of proinflammatory cytokines, and enhances apoptosis [46].

### 4.1. Conclusion

The protective effect of the corm of *Ensete ventricosum* (Welw.) Cheesman extracts at dose 400 mg/kg is comparable with the hepatoprotective effect of the silymarin which is standard hepatoprotective. Therefore, the finding of this study, along with the above facts, strongly suggests that hydromethanolic extract of the corm of *Ensete ventricosum* (Welw.) Cheesman has hepatoprotective properties, which are mediated by perhaps due to their phytochemicals property.

## Figures and Tables

**Table 1:** The result of preliminary phytochemical screening of hydromethanolic extract of the corm of *Ensete ventricosum* (Welw.) Cheesman.

Phytochemical constituent	Result	Phytochemical constituent	Result
Alkaloid	+	Quinone	+
Terpenoid	−	Saponins	+
Flavonoid	+	Tannin	+
Phenol	−	Glycosides	+
Steroid	+		

“+” stands for the presence of phytochemicals; “−” stands for the absence of phytochemicals.

**Table 2:** Effect of corm of *Ensete ventricosum* (Welw.) Cheesman extract on liver enzyme parameters and serum total bilirubin (TBIL) in isoniazid and rifampicin challenged mice.

Groups	ALT (IU/L)	AST (IU/L)	ALP (IU/L)	TBIL
Group I (normal control)	34.66 ± 3.42^a^	53.67 ± 4.40^a^	98.33 ± 2.49^a^	0.12 ± 0.01^a^
Group II (INH and RIF)	123.67 ± 3.25^d^	152.83 ± 3.84^d^	171.00 ± 4.69^c^	0.28 ± 0.02^b^
Group III (extract 200 mg/kg)	90.00 ± 4.89^c^	87.50 ± 4.96^c^	120.83 ± 3.75^b^	0.21 ± 0.03^ab^
Group IV (extract 400 mg/kg)	53.17 ± 4.95^b^	75.33 ± 3.21^bc^	108.00 ± 4.09^ab^	0.19 ± 0.03^a^
Group V (silymarin control)	40.16 ± 3.09^ab^	62.17 ± 1.62^ab^	106.33 ± 2.40^ab^	0.13 ± 0.01^a^

The results are expressed as mean ± SE. Values with different superscripts within the same column are statistically significant (*p* < 0.05).

**Table 3:** Liver index in different experimental groups of mice.

Groups	Liver index (L/BW %)
Group I (normal control)	5.85 ± 0.30^a^
Group II (INH and RIF)	7.54 ± 0.23^c^
Group III (extract 200 mg/kg)	6.89 ± 0.20^bc^
Group IV (extract 400 mg/kg)	6.14 ± 0.17^ab^
Group V (silymarin control)	6.00 ± 0.25^ab^

The results are expressed as mean ± SE. Values with different superscripts within the same column are statistically significant (*P* < 0.05).

## Data Availability

The data used to support the findings of this study are available from the corresponding author upon request.
